# Investigating the Reasons behind Companion Animal Relinquishment: A Systematic Content Analysis of Shelter Records for Cats and Dogs, 2018–2023

**DOI:** 10.3390/ani14172606

**Published:** 2024-09-07

**Authors:** Michael A. Kisley, Esther J. Chung, Hannah Levitt

**Affiliations:** Department of Psychology, University of Colorado, Colorado Springs, CO 80918, USA

**Keywords:** animal relinquishment, animal surrender, companion animals, cats, dogs, behavioral issues, content analysis

## Abstract

**Simple Summary:**

This study investigates the reasons behind the relinquishment of companion animals, specifically cats and dogs, to an open-admission shelter in the US. We analyzed 2836 randomly selected owner relinquishment records over a six-year period (2018–2023) using content analysis, a systematic method for categorizing qualitative data. The most common reasons identified for relinquishment included behavioral issues (28%), housing/moving (18%), and the inability to care for the animals (16%). Aggression, social conflict, and soiling were the most common behavioral-issue reasons reported. However, differences were observed in the pattern of relinquishment reasons based on statistical analyses of species, type of relinquishment, and year. The study found no significant increase in relinquishments due to financial or housing pressures during the peri-pandemic period, but there was an increase in relinquishments due to owners being otherwise unable to care for their pets in 2022 and 2023. This research provides valuable insights for animal welfare organizations and policymakers to develop strategies to better understand animal relinquishment and improve the welfare of both animals and their owners.

**Abstract:**

The relinquishment of companion animals to shelters presents significant challenges for animal welfare organizations and has substantial implications for the well-being of both the animals and their owners. This study aims to investigate the reasons for voluntarily relinquishing animals to shelters, to examine species-specific differences (cats and dogs), to compare initial relinquishments to returns (re-relinquishments or readmissions), and to identify potential changes over a 6-year period framing the onset of the COVID-19 pandemic. A retrospective analysis of owner relinquishment records from an open admission shelter in the US was conducted using content analysis, a novel approach in this area of research. A total of 13 relinquishment reason categories were identified, as well as 9 separate categories for specific behavioral issues. Among 2836 randomly selected records, the most frequent reasons cited were Behavior Issues (28%), Housing/Moving (18%), Unable to Care (16%), Too Many Pets (10%), Financial (6%), and Owner Allergies (5%). The most common behavioral reasons reported were Aggression (32% of behavioral surrenders), Social Conflict (28%), and Soiling (13%). However, differences were observed in the pattern of relinquishment reasons based on statistical analyses of species, type of relinquishment, and year. Regarding temporal trends, Housing/Moving and Financial reasons were not found to have changed significantly since the pandemic, but the relative frequency of the category Unable to Care increased significantly in 2022 and 2023. Collectively, these findings partially replicate those from research spanning the past several decades in this area that has employed less systematic methodology and can further be used to help identify and understand the primary drivers of owner relinquishments.

## 1. Introduction

The relationship between humans and non-human animals, including companion animals, has become an integral component of society and an active area of investigation across multiple scientific fields [1,2,3,4,5]. As the number of companion animals increases, the importance of understanding human–animal relationships grows. This understanding is crucial not only for the welfare of these animals but also for the well-being of their human counterparts and society as a whole [6,7,8,9]. One aspect of this intricate relationship between humans and their companion animals that has attracted attention is the voluntary relinquishment of animals to shelters, which places burdens on the animals, the shelters, and society more broadly [10,11]. The significance of this issue is underscored by the substantial number of animals that are relinquished to shelters each year: between 1.7 and 2.0 million cats and dogs in the US alone during the period of investigation for the present study (2018–2023) [12]. This speaks to the importance of research into the reasons behind relinquishment to foster a better understanding of this phenomenon and further develop strategies for management and potential interventions, e.g., [13,14,15]. We decided to exclusively employ the term relinquishment instead of surrender throughout this paper. Although there is some disagreement on the functional definition of surrender (compare [10] to [16]), the term relinquishment appears to be broadly understood to imply the act of voluntarily giving up an animal to another person or, in this case, to an animal shelter.

Consideration of the existing literature on the reasons for animal relinquishment reveals a diversity of study designs and methodologies and a corresponding heterogeneity of findings that are challenging to combine into a cohesive picture [10,11,17]. Some researchers have approached the issue with standardized questionnaires administered to pet owners at the time of relinquishment or soon thereafter. Miller et al. [18] reported that an owner’s housing move was the most common reason for cat relinquishment (29%), followed by owner illness (15%) at a local humane society in the US. For dogs, animal behavior was most prevalent (30%), followed by time or money limitations (21%). Diesel and colleagues [19] studied reasons for relinquishment across multiple shelters in the UK. They found problematic behaviors to be the most common reason (34%), followed by “needs more attention than can be given” (28%). Analysis of existing shelter records represents another approach to investigate reasons for voluntary animal relinquishment. A study of cat relinquishments at a shelter in Australia highlighted “too many pets/financial” as the top reason (27%) [20]. In another Australian study of cat relinquishments to multiple shelters, “pets not allowed” topped the list of reasons (21%), followed closely by too many pets (18%) [21]. Yet another approach has been to engage owners through a semi-structured interview process. Scarlett et al. [22] found that owner’s “health and personal issues” were the most common reasons for cat relinquishments in the US but only the third most frequent for dogs, after behavioral issues and housing concerns. Weiss and colleagues [15] found that “factors unrelated to the dog”, primarily housing-related issues, were most common among owners relinquishing their large-breed dogs to two shelters in large US cities. In a sample drawn from a low socioeconomic region of a large US city, the cost of medical care for animals was the most commonly cited reason for dog relinquishment [23]. As apparent from this brief review, leading reasons for animal relinquishing vary greatly from study to study, presumably depending upon multiple factors.

It is difficult to determine which elements (e.g., methodology employed, species studied, geographic region) may contribute to the variability in these findings because the field lacks a formalized, systematically determined set of relinquishment reason categories that all researchers use [10,11]. The majority of studies do not mention how relinquishment reason categories were determined or how individual owner responses were assigned to a given category. Those that do try to explain how categories were formulated typically refer to one or more of the following: consideration of past published research, e.g., [11,17], the expertise of the researchers, e.g., [24], the availability of specific socioeconomic indicators in the public domain, e.g., [25], or consultation with shelter professionals, e.g., [14]. Though certainly appropriate, the potential for variability between studies with such approaches is high, and this poses a substantial challenge to being able to compare results across studies. The present investigation employs a novel approach in this area of research, which is to use a content analysis approach with naïve but trained raters to (a) determine appropriate categories to use for owner relinquishment reasons and (b) assign each individual owner relinquishment record to one of those categories. Content analysis is a systematic, replicable technique for collapsing information such as textual narratives into categories for the purposes of characterizing the information and conducting subsequent quantitative analyses [26,27], and is only just beginning to be applied to the study of animal welfare and human–animal relationships, e.g., [28].

In addition to introducing a content analysis of animal relinquish reasons, the research described here was designed to address three specific questions regarding variation in those reasons in order to deepen our understanding of this aspect of the relationship between humans and their companion animals. First, very few comparisons have been made between the reasons why people relinquish dogs and cats. Some studies focus only on one or the other. However, even those that do investigate both species rarely make direct, statistical comparisons. Second, it would appear from existing literature that the reasons why people return animals after an adoption (variously referred to as “re-relinquishments” or “readmissions”) may differ from the reasons why people relinquish an animal that was not originally adopted from the shelter. A review of the existing literature suggests that an animal’s behavior may play an especially important role in returns, although findings vary. Mundschau and Suchak [17] reported animal behavior as the most common reason for cat returns (26%), followed by the owner’s “personal” reasons (14%). Powell et al. [29] found that the most common reasons why owners return animals after adoption are related to the animal’s behavior and social conflict (“incompatibility”) with other pets in the home, accounting for >50% of dog returns and >40% of cat returns. However, Hawes and colleagues [30] cited “personal reasons” as the most common reason for cat returns, though behavioral issues still represented the most frequent reason for dog returns. Unfortunately, no past studies we found include both initial relinquishments and returns in order to allow a direct, statistical comparison. Typically, existing publications describe one or the other type of relinquishment. Whether reasons for relinquishment differ statistically between animals’ first relinquishment and return after adoption is the second main question addressed by this study.

A third and final concern of this study relates to the possibility that the primary reasons behind voluntary animal relinquishment could change over time due to geopolitical, economic, cultural, or other reasons. This has rarely been directly investigated, and not recently that we are aware of. For example, a study of a large Danish shelter found owner health issues to be the most common reason for relinquishment of dogs and cats, with no detectable “linear changes” in that or other reasons cited from 1996 to 2017 [24]. However, the use of a linear model limits the ability of the researcher to detect potential non-linear events. Rapid changes in societal norms and pressures, including recent ones such as those induced by the COVID-19 pandemic, as well as recent financial and housing instabilities, necessitate an updated examination of potential temporal shifts in the reasons behind animal relinquishment. As such, we employed categorical comparisons between years for the frequency of cited reasons why people relinquish animals over a time period bracketing the pandemic and associated societal shifts. Although we did not make *a priori* predictions for this study, the possibility for changes in relinquishment reasons is raised by the observation of shifts over recent years in human–pet relationships [31,32] and in the number of intakes and pattern of outcomes at shelters [12,33,34]. Although there has been scientific investigation of reasons for owner relinquishment during peak COVID-19 pandemic years [35,36], potential *changes* in reasons from the pre-through-post-pandemic timeframe has not yet been undertaken.

## 2. Materials and Method

### 2.1. Shelter, Species, and Time Frame

Owner relinquishment records were retrospectively obtained from the Humane Society of the Pikes Peak Region (HSPPR), an open-admission shelter with 2 locations serving primarily 2 metropolitan areas in Colorado, USA: the Colorado Springs area with a population of approximately 750,000, and the Pueblo area with a population of approximately 170,000 in 2024. For the purposes of the present study, we collapsed relinquishments and data from across the two locations, effectively conducting the analyses as if for a single shelter. HSPPR adheres to the principles of the Asilomar Accords of statistical transparency and reporting [37], and all data, including but not limited to intakes, adoptions, euthanasia, and transfers, are made available to the public (hsppr.org/about-us/public-information/, accessed on 1 July 2024). This shelter was selected for convenience, as the first author had already established a relationship with the organization. HSPPR provided permission for access to the records, and ethical approval was granted by the Institutional Review Board of the first author’s university.

For the period investigated, 2018–2023, between approximately 20,000 and 30,000 animals entered the shelter each year, including dogs, cats, and all other species combined, as a result of owner relinquishments, stray retrievals, animal law enforcement actions, and transfers from other shelters. In terms of owner relinquishments only for the animal species of concern for the present study, an average of 3109 dogs and 3357 cats entered the shelter each year during this 6-year period. A detailed breakdown of owner relinquishments is shown in Table 1. We decided to focus only on cats and dogs for the present study because they represent the largest proportion of animals entering the shelter, and collectively, they are the most impactful to the shelter’s operation. Further, most extant literature on relinquishment is concerned with one or both of these species in particular. We selected the period 2018–2023 because it represents the most recent data and also because it brackets the COVID-19 pandemic and associated societal disruptions, as well as financial and housing strains that have characterized recent years in the US, including in these metropolitan areas.

### 2.2. Relinquishment Records

When an owner voluntarily relinquished an animal to HSPPR during the period of analysis, an electronic record was created that included the relinquishment date, type (either first relinquishment of an animal or a voluntary return of an animal that had been adopted previously from HSPPR), species, and reason. Owners reported the reason for relinquishment verbally, and shelter staff typed it into the record as an open field entry. There was also a drop-down menu for relinquishment reasons, but we did not analyze that field because supervisors at the shelter reported that staff struggled to appropriately assign reasons to these categories in the moment, as evidenced by a greater than 80% frequency assignment to a general, catch-all category termed “personal reasons”. Additional information recorded that was not relevant to the research questions of the present study and, thus, not analyzed here includes shelter location (Colorado Springs or Pueblo), postal code of owner’s residence, whether the animal was microchipped, any known health issues, and other information.

All entries determined to be pertinent for the planned research by the shelter staff were provided to the researchers. Because content analysis is very time-intensive, we could not realistically analyze the many thousands of entries shared. Instead, we decided to closely investigate at least 400 records per year. This sample size was not based on a formal power analysis, which is a statistical method used to determine the minimum sample size needed to detect an effect with a specified level of confidence. Because we did not know before the content analysis how many relinquishment reason categories would be determined, we could not perform such an analysis a priori. However, rough guidelines have been advanced for a *χ*^2^ test, which is the statistic used for this study. According to Cochran [39], aiming for at least 10 records per category (in this case, reasons for relinquishment) should generally provide adequate power. By reviewing past literature, we found that 20 relinquishment categories provided a reasonable upper limit, suggesting a sample size of 200. However, to be conservative, we decided to double this and aim for a sample size of at least 400 records per year. Since some entries included in the records were not actual relinquishments but instead transcripts of phone calls between shelter staff and animal owners, we decided to randomly select 600 records from each year, hoping that at least 400 of those records would be actual relinquishments that could be analyzed. This goal was met (see Table 1). The number of entries analyzed varied from 419 to 496 per year, with an average of 472.67 (*SD* = 29.02). The proportion of analyzed entries was relatively balanced between dogs and cats. Dogs represented 49% of all analyzed entries. This value ranged across years between 47% and 51%. Overall, the analysis presented here represents 7% of all owner relinquishments to the shelter during the period from 2018 to 2023.

### 2.3. Content Analysis

We employed formal content analysis to (a) determine what categories would be appropriate for animal relinquishment reasons and (b) which category each individual relinquishment record should be assigned to. Content analysis is a well-established research technique that allows for the reduction of information, such as the owner relinquishment narratives recorded in the records described above, into relevant categories for the purposes of comparative and quantitative analyses [26,27]. None of the individuals who participated in the development of the coding process were animal welfare professionals or researchers with in-depth knowledge of the existing literature on animal relinquishment studies. This was a deliberate research design choice because we did not want historical or pre-conceived ideas to influence the development of the categories or the assignment of return reasons to those categories (following the guidance of [40,41]).

Initially, hundreds of randomly selected owner relinquishment records were reviewed by 3 individuals who met on multiple occasions to discuss the major themes that appeared to recur. This led to the development of a pilot codebook of relinquishment reason categories. A codebook, or coding scheme, is a document that consists of codes or categories (e.g., “financial reasons”, “animal behavior”) with brief explanations of each code, along with examples [26,41]. This is eventually used by coders or raters, who are the individuals who utilize the codebook to assign each record to one of the categories. The categories must be mutually exclusive and non-overlapping, in order that each record can be assigned to one, and only one, category [27]. The appropriateness of the pilot codebook was tested by the same 3 individuals along with 4 additional naïve coders on hundreds of records to determine that (i) the vast majority of records examined could be assigned to a unique category, (ii) the categories were clear and mutually exclusive, and (iii) naïve coders could successfully use the codebook to consistently and reliably assign owner relinquishment reason categories. The latter was assessed by relative cross-coder agreement. This process was repeated after two rounds of codebook revisions, until it was determined that the vast majority of records considered could be uniquely categorized and cross-coder consistently was high (formal cross-coder reliability analyses were conducted on the final dataset, as described below). The records originally employed for developing the codebook were not analyzed further and are not included in the analyses presented below.

Once the codebook was finalized, coding the randomly selected records for analysis in the present study was undertaken in a two-step process. In the first step, coders read the reason for relinquishment field and determined a code from 1 to 13 (codes and corresponding categories are provided in Table 2). There is no inherent meaning to the order of the relinquishment codes. The numbering reflects the order in which the categories were identified and eventually finalized by the research team. In the event that the initial reason for relinquishment was 10. Behavior Issues, coders then proceeded to the second step of coding, which was to determine a behavioral code from A to J (codes and categories are elaborated in Table 3). For each record, coders assigned a category based only on the first reason for relinquishment mentioned. Coders were instructed to ignore all other record entries and to avoid trying to interpret or “go beyond” what was explicitly stated in the record.

Each record was evaluated individually by 4 coders, each of whom was trained by the authors on the use of the codebook. Before they began coding the data for this study, they practiced on 2 sets of 100 records, received feedback on their coding, and were given the chance to ask questions after each round. Training, practice, and having multiple coders are important components of effective content analysis [26,40,41]. For the final coding of analyzed records, inter-rater reliability was computed in order to evaluate consistency between raters. We determined Fleiss’ Kappa to be the most appropriate measure since we had more than 2 raters for each entry [41]. Using Cicchetti’s recommendations [38], a Kappa value of 0.75 or above was considered “excellent”. Final codes were determined by a majority rules rubric: whichever code received the most endorsements out of the 4 was assigned as the final code [42]. In cases for which there was a tie, the first author reviewed the record and made the final decision. This occurred in 6% of records analyzed.

### 2.4. Data Analysis

The reasons for relinquishment were analyzed in terms of proportions, where each category represented a percentage of all analyzed relinquishments. This approach enabled extrapolation from randomly selected records to estimate the distribution of reasons across all relinquishments to the shelter during the specified time period. Because the data were categorical, we utilized *χ*^2^ tests, a non-parametric statistical method designed specifically to assess associations between two or more categorical variables [43]. Given that the data consisted of nominal categories without intrinsic order or ranking, the *χ*^2^ test was the most appropriate analysis to determine if significant differences existed in the frequency of observed reasons. We also examined whether the distribution of proportions varied significantly across several variables: species (cat, dog), type (original relinquishment, return), and year (treated categorically to allow for detection of non-linear effects: 2018, 2019, 2020, 2021, 2022, 2023). Our data met fundamental statistical assumptions required for employing *χ*^2^ tests, including the use of mutually exclusive categories, which is an inherent aspect of content analysis. Because some analyses involved expected cell counts below 5, Monte Carlo exact tests were used to determine *p*-values. When significant associations were detected, post-hoc cell comparisons were conducted with *z*-scores following the guidance of Sharpe [44] and as implemented in SPSS v. 29.0, including Bonferroni correction for multiple tests.

## 3. Results

**Figure 1:**
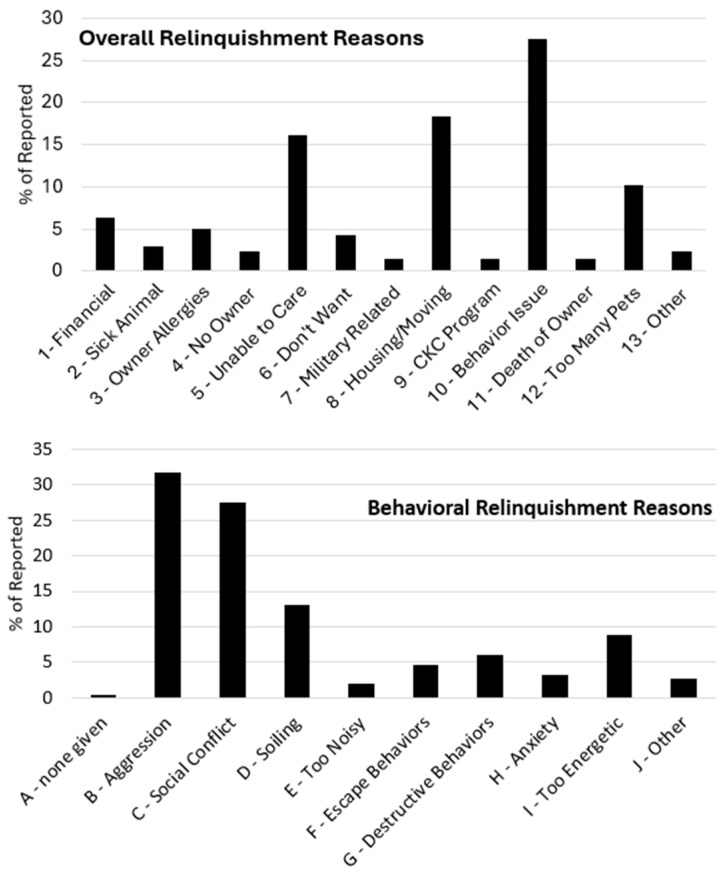
Proportion of overall relinquishment reasons (top panel, *N* = 2836) and behavioral relinquishment reasons (bottom panel, *N* = 782) for all records collapsed across species, type of relinquishment, and years. Only cases that were coded as 10—Behavior Issues during the first step of coding were subsequently coded with a behavioral reason. Reasons for relinquishment are listed according to the order from the content analysis coding keys, which are elaborated in Table 2 and Table 3.

The frequency of reasons cited for owner relinquishments varied based on the specific reason given. The proportion of reasons cited by owners upon relinquishing animals for all analyzed entries from 2018 to 2023 combined (*N* = 2836) is shown in the top panel of Figure 1. A *χ*^2^ test for goodness of fit was significant, *χ*^2^= 2956.96, *df* = 12, *p* < 0.001, indicating that the distribution of reasons differed from a uniform distribution (which would correspond to all reasons cited a statistically indistinguishable proportion of the time). Numerically, looking at reasons that were cited more than 5% of the time, Behavior Issues was most frequent (28%), followed by Housing/Moving (18%), Unable to Care (16%), Too Many Pets (10%), Financial (6%), and Owner Allergies (5%). For all entries that cited Behavior Issues as the initial reason (*N* = 782), an additional analysis was performed, and a corresponding distribution is shown in the bottom panel of Figure 1. This distribution was also different from uniform, *χ*^2^ = 851.63, *df* = 9, *p* < 0.001. Aggression was numerically the most frequently cited behavioral reason (32%), followed closely by Social Conflict (28%). Other behaviors cited at least 5% of the time when Behavior Issues was the initial reason were Soiling (13%), Too Energetic (9%), and Destructive Behaviors (6%).

### 3.1. Comparison of Species: Cats vs. Dogs

**Figure 2:**
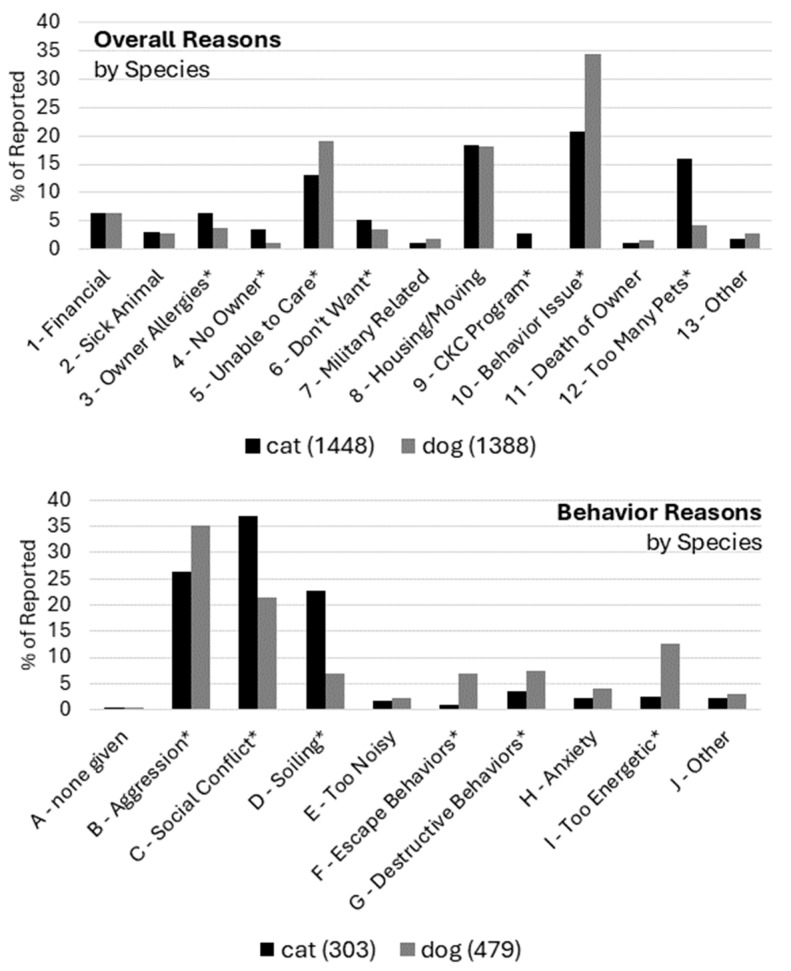
Proportion of overall (**top** panel) and behavioral (**bottom** panel) relinquishment reasons for all records, broken out by species. Codes with an asterisk (*) indicate significant post hoc, Bonferroni-adjusted comparisons between cats and dogs.

An association with animal species and reason for relinquishment was also detected in this sample. The distributions of overall reasons and behavioral reasons are shown in Figure 2. *χ*^2^ tests of independence were significant for both overall reasons, *χ*^2^ = 235.15, *df* = 12, *p* < 0.001, and for behavioral reasons, *χ*^2^ = 98.57, *df* = 9, *p* < 0.001, indicating that the distribution of reasons differed between cats and dogs. Post hoc, Bonferroni adjusted comparisons showed that cats and dogs differed on the frequency of a number of initial relinquishment reasons. Owner Allergies (6% cats, 4% dogs), No Owner (4% cats, 1% dogs), CKC Program (3% cats, 0% dogs), and Too Many Pets (16% cats, 4% dogs) were higher for cats. Unable to Care (13% cats, 19% dogs) and Behavior Issues (21% cats, 35% dogs) were higher for dogs. Looking at specific behavioral issues, Aggression (26% cats, 35% dogs), Escape Behaviors (1% cats, 7% dogs), Destructive Behaviors (4% cats, 8% dogs), and Too Energetic (3% cats, 13% dogs) were higher for dogs. Social Conflict (37% cats, 22% dogs) and Soiling (23% cats, 7% dogs) were higher for cats.

Taken as a whole, this pattern of findings reveals two important points. First, there was some commonality between cats and dogs in the stated reasons why people relinquished their animals. For example, Unable to Care, Housing/Moving, and Behavior Issues were frequent reasons for both species. Second, there were nevertheless notable differences between species. Too Many Pets stood out as a reason that was more frequent for cats, whereas Behavior Issues stood out for dogs. Examination of specific behaviors revealed several additional notable differences, including especially Soiling, which was higher for cats, to be contrasted with Aggression, which was higher for dogs. The observed significant species difference in the CKC Program was expected, as this program was only available for relinquishing cats.

### 3.2. Comparison of Type: First Relinquishment vs. Return

**Figure 3:**
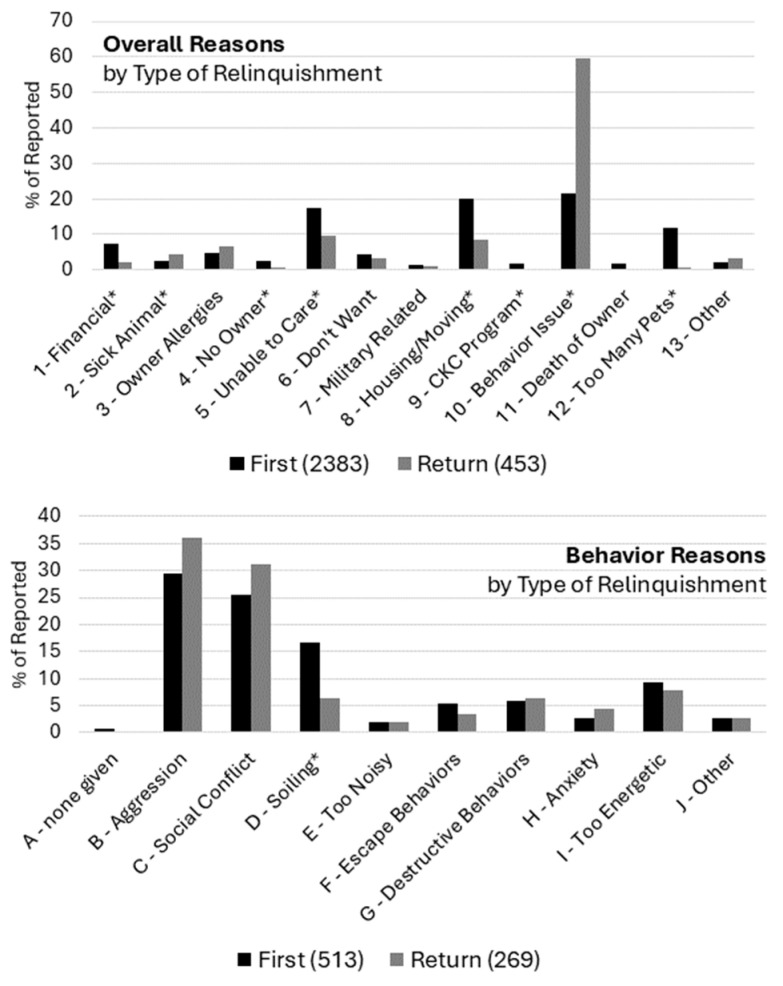
Proportion of overall (**top** panel) and behavioral (**bottom** panel) relinquishment reasons for all records, broken out by type of relinquishment. Codes with an asterisk (*) indicate significant post hoc, Bonferroni-adjusted comparisons between first-time and return relinquishments.

Reasons for relinquishment were also found to differ between animals who were relinquished to the shelter for the first time compared to animals who were relinquished more than once, so-called “returns” (Figure 3). *χ*^2^ tests of independence were significant for both overall reasons, *χ*^2^ =334.10, *df* = 12, *p* < 0.001, and for behavioral reasons, *χ*^2^ = 23.87, *df* = 9, *p* < 0.001. Post hoc, Bonferroni corrected comparisons showed that returned animals were associated with a higher proportion of Behavior Issues (22% first, 59% return) and Sick Animal (3% first, 4% return) reports compared to first-time relinquishments and a lower proportion for most other reasons including Financial (7% first, 2% return), No Owner (3% first, 1% return), Unable to Care (17% first, 10% return), Housing/Moving (20% first, 8% return), CKC Program (2% first, 0% return), and Too Many Pets (12% first, 1% return). Looking more closely at specific behavioral issues, only a difference in Soiling (17% first, 6% return) as a cited reason was detected, with first-time relinquishments showing a higher proportion of mentions compared to returns in this category. Broadly, these findings are consistent with the idea that Behavior Issues were a more common reason for return relinquishments (and accounted for more than half of returns) compared to first-time relinquishments, and most other reasons were, therefore, proportionally reduced in frequency.

### 3.3. Comparison of Years: 2018–2023

**Figure 4:**
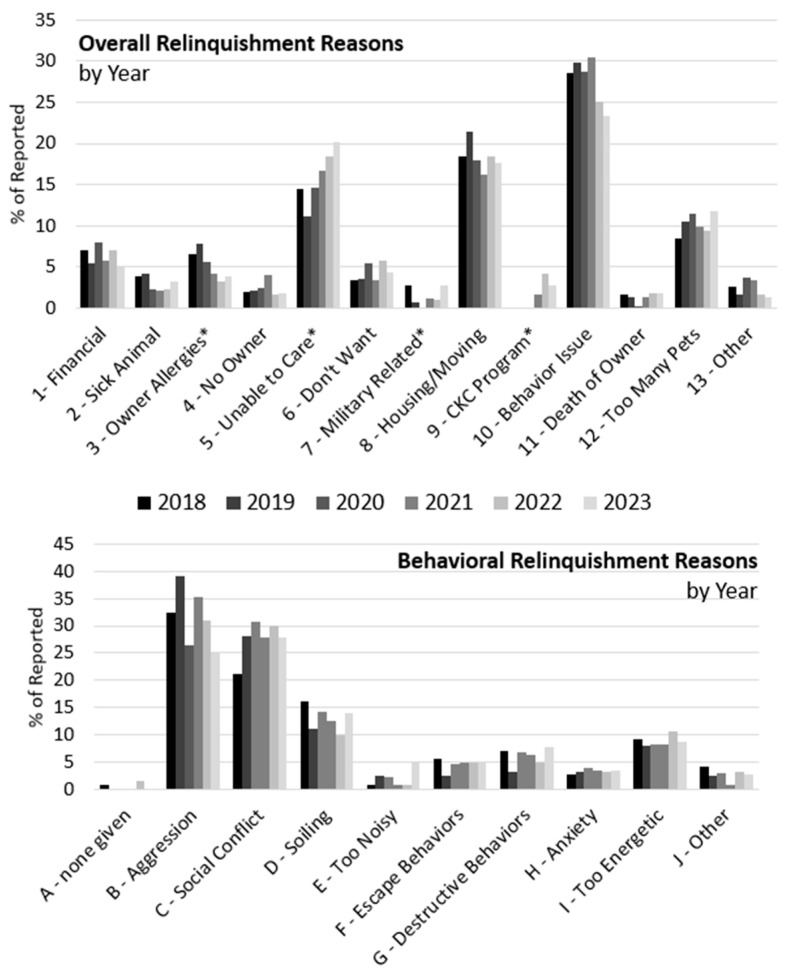
Proportion of overall (**top** panel) and behavioral (**bottom** panel) relinquishment reasons for all records, broken out by year. Codes with an asterisk (*) indicate the presence of at least one significant post hoc Bonferroni-adjusted comparison between years for that category. Specific significant pair-wise effects are reported in the main text.

Reasons for relinquishment were found to vary with year as well. However, the overall distribution of the most common reasons remained relatively stable (see Figure 4). For example, Behavior Issues, Housing/Moving, Unable to Care, and Too Many Pets were consistently the most commonly cited reasons, numerically. Nevertheless, an *χ*^2^ test of independence was significant for overall reasons, *χ*^2^ =154.14, *df* = 12, *p* < 0.001. Post hoc, Bonferroni corrected comparisons revealed significant differences in the frequency of the following reasons. For Owner Allergies, reports in 2022 (3%) were lower than in 2019 (8%). For Unable to Care, reports in 2022 (19%) and 2023 (20%) were higher than in 2019 (11%). For Military-Related, reports in 2020 (0%) were lower than in 2018 (3%) and 2023 (3%). For the CKC Program, reports in 2022 (4%) and 2023 (3%) were higher than in 2018 (0%), 2019 (0%), and 2020 (0%). This latter finding is to be expected, as the CKC program was not initiated until the middle of 2021. The distribution of behavioral reasons for relinquishment did not differ by year, as the *χ*^2^ test was not significant, *χ*^2^ = 37.50, *df* = 9, *p* = 0.79. This may have resulted from a relatively small number of cases per year, between 115 and 142. Based on observation of the distributions, Aggression and Social Conflict were consistently the most cited reasons, numerically, followed by Soiling and Too Energetic. Taken as a whole, these results suggest broad stability in the overall distribution of reasons for relinquishment over the time period investigated (2018–2023), although there were select changes across years that are discussed below.

## 4. Discussion

The present study employed content analysis to estimate the relative frequency of reasons why owners voluntarily relinquish their animals to a shelter. Because we engaged investigators without extensive knowledge of research in this area to develop the codebook and naïve coders to assign each record to a category, our approach can be seen as bottom-up and less susceptible to potential pre-conceived assumptions and biases regarding human–animal interactions, common reasons for relinquishment from past research, known differences between species, and the impact of societal trends on animal welfare. Evidence to support the effectiveness of our approach includes high inter-rater reliability (“excellent” according to Cicchetti [38]) and the low proportion of records that were coded as “other” (2%). The latter suggests that the categories developed here encompass the vast majority of owner-stated relinquishment reasons, and the former observation speaks to the appropriateness and separability of those reasons. Overall, the reason categories determined during the codebook development process here overlapped with categories that had been identified through less systematic methods in past research. For example, our initial relinquishment reason categories appear comparable to what Coe et al. [10] summarized from past literature as human-related reasons, including, notably, moving/housing, lack of time/responsibility, economic issues, health issues, allergies, and unwanted/too many animals. Our behavioral reason categories largely overlap with what has previously been described as animal-related reasons such as aggression, other social conflicts (sometimes referred to as “incompatibility” in other studies), destructiveness, noisiness, escape, and anxiety. As such, and despite differences in specific terms, category parsing, and interpretation, the results of the bottom-up approach employed here largely validate the more top-down, a priori-determined reasons identified in past research.

### 4.1. Finding Highlights

Animal behavior was the most commonly cited reason for relinquishment in the present study and represented more than a quarter of all relinquishments at the specific US shelters investigated over the period from 2018 to 2023. This is consistent with past literature for which behavior has consistently been highlighted as among the most common reasons for relinquishment [10,19,45,46]. Although helpful for understanding both cat and dog relinquishments, behavior was cited significantly more often for the latter in the present study. This pattern is also largely consistent with past literature [18,22], although we are not aware of a direct statistical comparison between species until now. When behavior was cited, the specific behaviors also differed between cats and dogs. Aggression was the most common behavioral reason for dog relinquishment and was significantly more common than for cats. Social conflict was the most common behavioral reason for cat relinquishment and was significantly more common than for dogs. This potentially reflects real species differences in behavioral responses to social others, although it may also partially arise from differences in how humans perceive and interpret the behavior of cats and dogs, e.g., [47,48]. Cats were relinquished more often for soiling, and dogs more frequently relinquished for escape, destructive, and anxious behaviors. These patterns are broadly consistent with owner complaints about pet behavior based on surveys of the owners themselves and of veterinarians [49]. It is worth reiterating here that this study, like most studies of animal relinquishment, is based on owner observations and, therefore, limited by the extent to which owners accurately perceive and understand animal behavior. Finally, the proportion of records involving citation of animal behavior was not found to differ significantly in pair-wise comparisons among the 6 years investigated, 2018–2023. Given the design of the present study, it is important to note that this does not preclude a change in the overall number of such relinquishments.

Behavior issues were substantially and significantly more frequently reported for returns (readmissions, re-relinquishments), accounting for nearly 60% of animals returned to the shelter after being adopted, broadly consistent with other studies [29,30,50]. This finding suggests that in-shelter behavior programs, post-adoption behavior assistance, or extending foster care stays may be helpful interventions for reducing return rates, also see [14,17,51,52]. Unfortunately, very few systematic intervention studies have been conducted to directly test for the potential beneficial impact of such interventions on return rates [53]. Diesel and colleagues did find that owners who sought behavioral support after adoption were less likely to subsequently relinquish their animals [50]. However, that was not a randomized intervention study, so it remains possible that those who sought help may have already been less likely to relinquish their pets. There is a need for much more research in this area, including targeted, controlled intervention studies, especially given the clear and highly replicated evidence for the role that behavioral issues play in returns after adoption. Further, it has been argued that a variety of different intervention modalities and approaches will need to be developed and tested in order to address the different needs, priorities, and resource limitations of owners from a diverse array of contexts and backgrounds [54].

Issues related to owners’ circumstances were also frequently reported at the time of relinquishment. Housing/Moving was numerically the second most common reason here (approximately 18%), and this is frequently cited in other studies as being particularly important, as well [10,15,55,56]. Interestingly, and despite anecdotal reports that housing challenges have contributed to more relinquishments in recent years, we did not detect a significant change in the citation of this reason for animal relinquishments here. This was also true for statements from owners regarding financial reasons for relinquishment. As such, and at least for the specific metropolitan areas investigated here, societal disruptions associated with the pandemic and recent economic conditions did not appear to lead to a measurable change in the relative proportion of housing- and financial-based animal relinquishments between 2018 and 2023 (but note that overall number of relinquishments has been going up, see Table 2). Nevertheless, housing and financial strains remain important barriers to maintaining pets for many people, see also [11,23,25].

We are not aware of any shelter-based studies of animal relinquishment that focused on changes across the peri-pandemic era to which we can directly compare our findings. However, at the height of the pandemic in late 2020 and early 2021, Carroll and colleagues [35] conducted a multi-national online survey of 3945 animal owners, a small percentage of whom had relinquished (<1%) or considered relinquishing (~4%) their animals. These owners selected reasons from a drop-down menu of options, and they could select as many reasons as applied. Whether due to real discrepancies in reasons for relinquishment or due to methodological differences, the percentages differ substantially between our studies. The top reason cited by Carroll et al. [35] was financial constraints at 44%, compared to only 8% in 2020 and 6% in 2021 for our study. The second top reason in Carroll’s study was health concerns, such as fear of a pet transmitting the coronavirus to the owner or a family member at 33%. Because we did not have a category for COVID-19-related health concerns, any such mentions would have been coded as “Other” in the present study, a category that only accounted for 4% of relinquishments in 2020 and 3% in 2021 in our study. Behavioral issues were comparable between studies at 31% for Carroll et al. [35] vs. 29% in 2020 and 30% in 2021 for our study. However, it is difficult to make a direct comparison between these studies because of several methodological differences. For example, Carroll asked for relinquishment reasons from both those who had actually relinquished animals (*N* = 21) and those who had only considered doing so (*N* = 168). By contrast, all of our participants (*N* = 464 in 2020; *N* = 474 in 2021) had actually relinquished their animals. Further, Carroll et al.’s participants voluntarily opted into the online study, and it is possible that many people who had relinquished an animal may have avoided such a study [31]. Additionally, participants in Carroll’s study were prompted to select reasons from a list of options and could select multiple responses, whereas owners for the present study provided a free-form explanation, which was subsequently categorized through content analysis to a single reason (the first encountered in the record). Relinquishments for Carroll’s study, whether accomplished or considered, included those to either a shelter or another person (e.g., a friend or relative), whereas all relinquishments in our study were to a shelter. The large discrepancy in findings between studies highlights the importance of considering the specific methodologies employed in animal relinquishment research, as argued above. Finally, Carroll et al. did not have data from the pre-pandemic period or from the years that followed the height of the pandemic (e.g., 2022 and 2023) to allow an assessment of potential changes in the reasons for relinquishment across this period. By contrast, the present study design allowed for such an analysis, but did not uncover evidence for substantial changes in the relative pattern of relinquishment reasons after the onset of the pandemic, with one notable exception which is discussed next.

Additional owner-related challenges, grouped here into the category Unable to Care, were also reported at a high rate, about 16%. Unlike housing and financial reasons, this category did exhibit a significant increase in 2022 and 2023 compared to pre-pandemic rates, accounting for roughly one in five relinquishments over the last two years of this study, which was nearly double the rate of reports for this reason in 2019. Although a somewhat diverse category (compared to “personal issues” from Coe et al. [10]), the common underlying theme of Unable to Care relinquishments is generally related to an owner’s circumstances, having either not enough time or energy to care for an animal. It may be that the increasing demands on people to return to the workplace and other away-from-the-home routines in the post-pandemic era has contributed to the increased frequency of this relinquishment reason, although we do not currently have direct data to support this idea. For example, such trends could lead to decreases in the amount of time owners have to spend with and care for their animals. Additionally, the emotional labor associated with returning to work might reduce owners’ energy, again negatively impacting their ability to care for their animals. If true, these factors could help explain the recent rise in employers’ attempts to entice and retain workers through pet-friendly policies and workplaces [57,58]. However, additional research will be required to develop a deeper understanding of the increase in owner-reported inability to care for their animals in recent years. For example, future studies of animal relinquishment reasons might involve the development of sub-categories of the Unable to Care category, similar to what was done here for Behavior Issues. Such extensions of this research may prove helpful for identifying potential interventions and supports for owners and their pets.

A number of additional findings were uncovered with regard to less frequently reported reasons for animal relinquishment that may nevertheless be pertinent to some researchers and animal welfare professionals. For example, the citation of Too Many Pets accounted for about 10% of all relinquishments analyzed in this study, and no effect of year was detected. Among other implications, this suggests that education and pre-adoption counseling regarding the number of animals in one’s household continue to be important. This reason was cited significantly more often for cat (16%) than dog relinquishments (4%), which is consistent with the observation that cat owners tend to keep more cats, on average, than dog owners keep dogs [59]. Further, the number of kitten litters within US households has historically been double that of puppy litters [60]. Owner allergies were reported significantly more often for cat (6%) than for dog relinquishments (4%). This may reflect different allergen sensitivity rates of humans between these species within the US, where this study was conducted [61]. We also uncovered suggestive evidence of a potential decrease in the proportion of relinquishments involving the report of owner allergies in recent years compared to pre-pandemic levels (specifically, 2022 was significantly lower than 2019). This could have resulted from improved personal hygiene practices post-pandemic, e.g., [62], although direct evidence linking this to reduced allergy symptoms is currently lacking. As another potential explanation, base population rates of allergic disease were actually found to decrease in several countries during and after the peak of the pandemic [63], though we did not find any such studies for the US. Although a relatively infrequent reason for relinquishment at only 1% overall, Military-Related reasons did show a noteworthy time-based effect. Specifically, this category accounted for 0% of analyzed relinquishments in 2020, which was significantly different from 2018 and 2023 (both 3%). This may be attributable to temporary changes in US Department of Defense policies during the pandemic, whereby travel, station changes, and other forms of relocation were restricted in response to the pandemic [64,65].

### 4.2. Limitations and Future Directions

Several additional study limitations should be considered to avoid over-interpreting these findings or applying them inappropriately. First, all reasons for relinquishment analyzed here were based on owner reports and, therefore, do not necessarily represent with accuracy the underlying reasons for relinquishment. Social desirability may be a particularly influential factor when people are asked why they are relinquishing their animal to the shelter [10,45]. Relatedly, owners tend to underestimate and underreport behavioral issues [66], suggesting that this category, which was already the most frequently reported reason for animal relinquishment here, may be even more common as an underlying reason. Nevertheless, we are not aware of any reasons why potential distortion of owner reports would differ systematically between cats and dogs, between initial relinquishments and returns, or across years studied. As such, and given the bottom-up study design employed here, in addition to the conservative statistical approach, the comparative differences in relinquishment reasons reported here are likely to be representative and interpretable. But one additional, related caution should be considered: reasons for relinquishment were categorized here based on brief narratives, which sometimes involved the statement of multiple reasons (e.g., “She was just too high energy for our household. Plus, I am very busy and didn’t feel like I had enough time to take care of her”). When this happened, only the first reason mentioned was coded (in this example, 10. Behavioral, 8. Too Energetic), and subsequent reasons remained uncoded (in this case, 5. Unable to Care). As such, the particular approach taken to content analysis here may neglect some level of complexity in the reasons for relinquishment provided by owners. Indeed, a detailed interview study provided evidence that the first reason stated may not always reflect the primary reason [24,45]. As such, future content analysis studies may allow the description of additional nuances by allowing for the coding of multiple reasons. Finally, by relying on pre-existing, shelter-based records, these data may be considered somewhat limited in the sense that they were not collected originally for research purposes [24].

Additional constraints imposed by our specific research design should be considered. The findings reported here depend, at least to some extent, upon the specific community and shelter contexts studied and may not be fully generalizable to other settings involving, for example, different levels of urbanization. To illustrate, Weiss et al. [15] reported personal and housing issues as the most common reasons for the relinquishment of dogs in larger US cities. Cultural differences may also be relevant, as Jensen et al. found that owner health was the most frequently reported reason for the relinquishment of dogs in Denmark, for example. However, as argued in the introduction, some level of disagreement between studies may also arise because of the different methodologies used. Applying the same categories described here with a consistent content analysis approach to data from other shelters would provide a way to more directly assess potential differences in the reasons why people relinquish their animals to shelters across different contexts. Future studies aimed at investigating differences in other aspects of individual human circumstances and psychology that relate to animal relinquishment may also be informative from both theoretical and applied perspectives.

## 5. Conclusions

In summary, this study highlights the multifaceted reasons behind the relinquishment of companion animals to shelters, emphasizing the significant role of animal behavioral issues and the varied nature of human-related factors such as housing, financial constraints, and other personal circumstances. A key contribution of this research is the novel application of content analysis, which provided a systematic and replicable method for categorizing relinquishment reasons. This approach allowed for an unbiased identification of the most frequent causes of animal relinquishment, largely validating and also extending findings from previous studies. Notably, our results underscore the need for targeted interventions aimed at addressing challenging behaviors, as these issues were particularly prevalent among returned animals, suggesting that post-adoption support and behavioral training could be helpful in reducing readmission rates. Furthermore, the increase in relinquishments due to the inability to care for animals in recent years points to the broader impacts of societal changes, potentially exacerbated by post-pandemic trends on pet ownership. However, at least for the metropolitan areas studied here, we did not see direct evidence that recent changes in financial and housing pressures were leading to elevated rates of animal relinquishment proportionally to other reasons. This study contributes to the existing literature by offering a comparative analysis of relinquishment reasons across species and time, thus providing a more nuanced understanding that can inform shelter practices and policies. Future research should continue to explore the underlying causes of animal relinquishment, particularly in different socioeconomic and cultural contexts, to develop more effective strategies for preventing unnecessary relinquishments and improving the welfare of both animals and their owners. More broadly, this line of research helps to inform a deeper understanding of the relationship between humans and their animal companions.

## Figures and Tables

**Table 1:** Number of Relinquishment Records by Year and Species.

	Dogs		Cats		Total		IRR	
Year	Reported	Analyzed	Reported	Analyzed	Reported	Analyzed	Overall Reasons	Behavior Reasons
2018	2934	243	2675	253	5609	496	0.80	0.76
2019	2932	223	3022	196	5954	419	0.77	0.76
2020	2245	219	2587	245	4832	464	0.82	0.80
2021	2707	222	3469	252	6176	474	0.82	0.84
2022	3604	229	4333	262	7937	491	0.83	0.83
2023	4234	252	4058	240	8292	492	0.83	0.79
**Total**	**18,656**	**1388**	**20,144**	**1448**	**38,800**	**2836**	**0.81**	**0.80**

Table Note: “Reported” corresponds to the total number of voluntary relinquishments for dogs, cats, and combined dogs and cats for the shelter for each year studied. “Analyzed” corresponds to the number of entries that were randomly selected for analysis here. IRR values are Fleiss’ Kappa inter-rater reliabilities, broken out by year, for all initial relinquishment reasons and for behavioral reasons. All values are “excellent” according to established criteria [38].

**Table 2:** Overall Relinquishment Reasons from Content Analysis.

Code	Category	Description
1.	Financial	Any mention of insufficient financial resources to care for an animal, including medical care, cost of food, or any other reason, as long as money was mentioned.
2.	Sick Animal	The owner cited the animal’s illness. If cost of care was mentioned, this would instead be coded as 1. Financial.
3.	Owner Allergies	Applied whether it was the owner themselves or other family members experiencing allergy symptoms due to the animal.
4.	No Owner	The individual relinquishing the animal either found the animal or received it from another person or source, believing the animal was without an owner.
5.	Unable to Care	The owner felt unable to care for the animal for any reason except financial. Common reasons included lack of time, being away from home too much, major life changes (not financial or housing-related), or a desire for the animal to have a better quality of life.
6.	Do Not Want	The owner did not mention any challenges to caring for the animal but did not want the animal nevertheless.
7.	Military-Related	When the stated reason was related to military issues such as transfer or deployment.
8.	Housing/Moving	The owner’s housing situation had changed in some way that impacted the animal, but not military-related. Common reasons included moving, breed restrictions, new landlord rules at the owner’s rental property, or becoming unhoused.
9.	CKC Program	The Community Kitten Care (CKC) Program launched in 2020 at the Pueblo location, allowing community members providing foster care to previously feral kittens to relinquish the animals once they were 8 weeks and 2 pounds. This is separate from the animal foster program that spans both locations.
10.	Behavior Issues	Selected if the animal’s behavior was the first reason cited. A subsequent behavioral code was then selected in a second step (see Table 3).
11.	Death	The passing of the owner, a family member, a significant other, or someone close.
12.	Too Many Pets	The stated reason included mentioned too many pets, the need to reduce the number of animals, unplanned litter, or other reasons for inability or lack of desire to care for so many animals. If money was mentioned, this was instead coded as 1. Financial.
13.	Other	Any stated reason that did not fit with one of the above categories or was too vague or unclear to be coded.

**Table 3:** Behavioral Relinquishment Reasons from Content Analysis.

Code	Category	Description
A.	None given	The owner did not provide any details beyond mention of the animal’s behavior.
B.	Aggression	Any mention of hostile behavior, including but not limited to causing harm to humans or other animals. Frequent behaviors mentioned included growling, biting, scratching, and any behaviors that were perceived as ‘scary’, ‘intimidating’, ‘reactive’, ‘too rough’, or similar.
C.	Social Conflict	Non-aggressive behaviors that nevertheless were perceived as arising from conflict with other animals or humans. This included showing fear of others, ‘not liking’ or ‘not getting along with’ others.
D.	Soiling	Animal eliminated outside of desired areas.
E.	Too Noisy	Noise made by the animal was specifically mentioned. This usually included barking, but sometimes cat mewing as well.
F.	Escape Behaviors	The primary reason cited related to the animal attempting to break free from living space. Common behaviors included running through open doors and jumping fences.
G.	Destructive Behaviors	The animal was causing damage to property.
H.	Anxiety	The animal showed behavior that can be classified as nervous, worried, or tense, including separation anxiety. If fearful behavior was induced by the presence of another animal, that would have been coded as 2. Social Conflict.
I.	Too Energetic	The animal showed great energy or vitality. Common citations typically included ‘too much’ energy, strength, or play behaviors.
J.	Other	Any stated reason that did not fit with one of the above categories or was too vague or unclear to be coded.

Table Note. Behavioral relinquishment reasons were only specified in a second step for records that were coded as 10. Behavior Issues in the first step of coding.

## Data Availability

The minimal data file can be accessed at https://osf.io/wuf2c/ (accessed on 1 July 2024).
